# Factoring economic costs into conservation planning may not improve agreement over priorities for protection

**DOI:** 10.1038/s41467-017-02399-y

**Published:** 2017-12-21

**Authors:** Paul R. Armsworth, Heather B. Jackson, Seong-Hoon Cho, Melissa Clark, Joseph E. Fargione, Gwenllian D. Iacona, Taeyoung Kim, Eric R. Larson, Thomas Minney, Nathan A. Sutton

**Affiliations:** 1 Department of Ecology and Evolutionary Biology, University of Tennessee, Knoxville, TN 37996 USA; 2 Department of Agricultural and Resource Economics, University of Tennessee, Knoxville, TN 37996 USA; 3 The Nature Conservancy, 99 Bedford Street, 5th Floor, Boston, MA 02111 USA; 4 The Nature Conservancy, 1101 West River Parkway, Suite 200, Minneapolis, MN 55415 USA; 5 ARC Centre of Excellence for Environmental Decisions, University of Queensland, St Lucia, QLD 4072 Australia; 6 Department of Food and Resource Economics, Institute of Agriculture and Life Sciences, Gyeongsang National University, JinJu-si, Gyeongsangnam-do 52828 Republic of Korea; 7 Department of Natural Resources and Environmental Sciences, University of Illinois, Urbana, IL 61801 USA; 8 The Nature Conservancy, 435 Wilson Street, Elkins, WV 26241 USA

## Abstract

Conservation organizations must redouble efforts to protect habitat given continuing biodiversity declines. Prioritization of future areas for protection is hampered by disagreements over what the ecological targets of conservation should be. Here we test the claim that such disagreements will become less important as conservation moves away from prioritizing areas for protection based only on ecological considerations and accounts for varying costs of protection using return-on-investment (ROI) methods. We combine a simulation approach with a case study of forests in the eastern United States, paying particular attention to how covariation between ecological benefits and economic costs influences agreement levels. For many conservation goals, agreement over spatial priorities improves with ROI methods. However, we also show that a reliance on ROI-based prioritization can sometimes exacerbate disagreements over priorities. As such, accounting for costs in conservation planning does not enable society to sidestep careful consideration of the ecological goals of conservation.

## Abstract (web summary)

Prioritising areas for conservation is hindered by disagreements over ecological targets. Here, Armsworth et al. combine a simulation approach and case study to test if considering economic return on investment aids in prioritisation, and find that its impact on reaching agreements varies greatly.

## Introduction

Faced with ongoing declines in biodiversity and many ecosystem services^1,2^, governments and societies around the world are seeking to expand the coverage of protected areas^3^. Yet resources to establish new protected areas are limited^4,5^ and must be allocated effectively^6,7^. Efforts to prioritize locations for future protection are hampered by disagreements within and between conservation organizations over just what the targets of conservation should be^8–12^. Should we prioritize areas for protection that would safeguard important ecological functions or those that protect the most species? If emphasizing species, what taxonomic groups should we focus on? And how much weight should we give to covering more species vs. ensuring persistence of those that already occur on protected sites? Even if conservation targets can be agreed upon, there are still many ways to estimate the contribution that protecting a given site would make to advancing conservation of those targets. What ecological indicators should be used? How should they be measured and over what scales? Just what targets are chosen and how progress towards these targets is measured determines the set of priorities recommended for future protection^13–18^.

Software and other tools used to inform spatial prioritization decisions in conservation are growing in sophistication^19^, as are attempts to evidence their influence on actual land protection decisions^20–22^. Alongside ecological considerations, planning tools now often include other factors important to conservation decision-making such as the cost of protecting different sites^23^, the threats impacting them^24^, the feasibility of working in different environments^25^, and the scope for leveraging support from local communities for protection efforts^26^. Among these factors, we focus on the economic cost of protecting different sites. Early spatial prioritization efforts assumed conservation activity was limited by the total amount of land set aside^27,28^. But, often, financial support for conservation is more restrictive and greater progress towards conservation goals can be made if spatial variability in economic costs is accounted for in planning^29^. Therefore, over the past two decades, conservation planning approaches have increasingly focused on identifying locations for protection that will be both ecologically effective and cost effective^29–32^. When accounting for the cost of protecting sites, conservation organizations often prioritize locations based on the return-on-investment (ROI) they offer. The simplest version of ROI is simply the ecological benefit offered by protecting a site divided by the cost of achieving that protection. Locations offering large conservation gains per dollar provide good candidates for future protection.

Disputes over what aspects of biodiversity to target for protection and over how to quantify progress towards conserving those targets might become less important once costs are accounted for in ROI-based planning. Bode et al.^33^ first proposed this idea, which has been repeated in major conservation textbooks^34,35^. Bode et al.^33^ argued that spatial variability in the cost of protecting sites was sufficient to dominate the variation in ecological benefit metrics that one might use to prioritize locations for protection, resulting in greater agreement over future priorities. Related claims have since appeared in other studies^36–39^.

Any test of whether accounting for economic costs improves agreement over future conservation priorities needs to preserve patterns of covariation and relative variability between the economic cost and ecological benefit data used. Cost estimates need to be similar to those that conservation organizations actually confront. Also the spatial grain of the ecological benefit and economic cost data must be closely matched. Unfortunately, many conservation planning studies fail to meet these standards^23,29^.

**Fig. 1:**
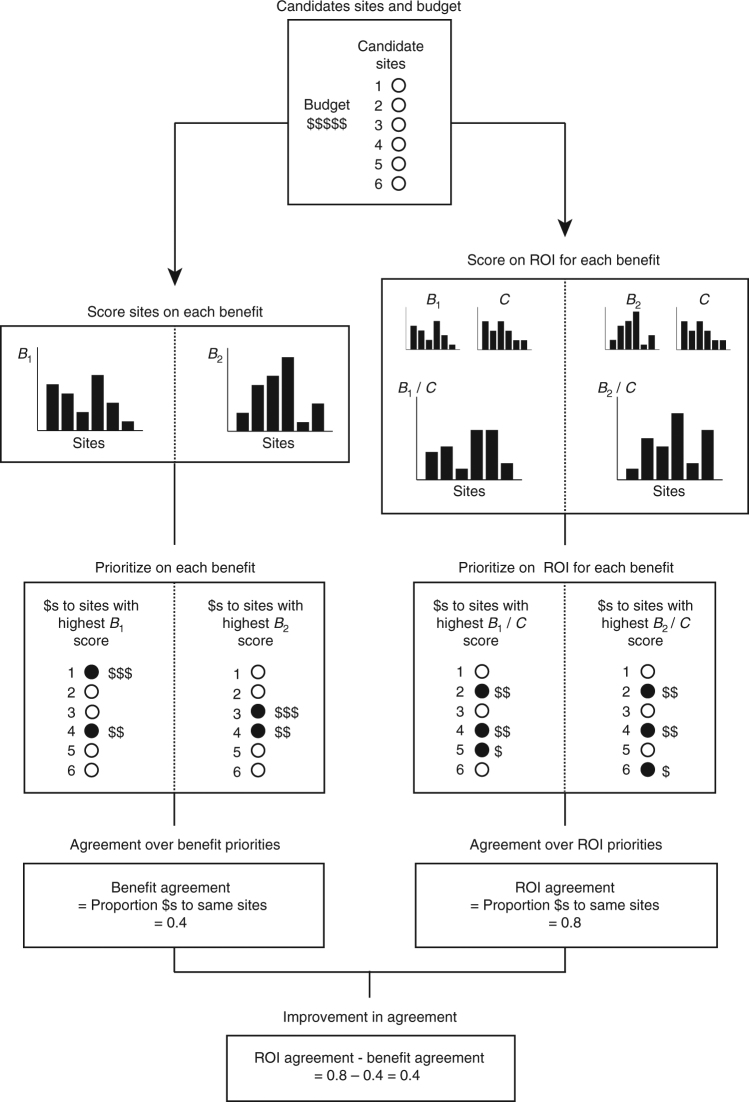
Illustration of the steps in the analysis. Once a set of candidate sites for protection and budget level are established, candidate sites are ranked for protection using two different ecological benefit metrics *B*_1_ and *B*_2_ and the agreement level between the two prioritizations is calculated as the share of the budget allocated to the same set of sites. This process is repeated when ranking sites for protection using conservation return-on-investment, *B*_1_/*C* and *B*_2_/*C*, respectively. Finally, the levels of agreement between the benefit-only prioritizations and the ROI-based prioritizations are compared

We test the claim that moving to ROI-based planning in conservation will result in greater agreement over future priorities. First, we determine agreement levels over priority locations for protection that would result if relying on different ecological benefit metrics (*B*_1_ and *B*_2_ in Fig. 1), referred to as Benefit Agreement throughout. We then examine agreement levels over protected area priorities when also accounting for economic costs using an ROI framework (prioritizing based on *B*_1_/*C* and *B*_2_/*C* in Fig. 1); we refer to this as ROI Agreement. We develop hypotheses regarding when a particular large improvement in agreement levels over priorities can be expected when shifting to ROI-based prioritization and test them by combining a simulation approach with a case study application. The case study data come from field surveys and geographic information system (GIS) analyses of protected areas acquired to conserve forest ecosystems in the central and southern Appalachian Mountains. Many endemic species having only a small proportion of their ranges currently protected in the United States are concentrated in these ecosystems^40^. The region also has a particularly important role to play in enabling species movements under climate change^41^. For our case study, we focus on areas acquired for protection in the region between 2000 and 2009 by The Nature Conservancy (TNC), a nonprofit land trust^42,43^.

In both the simulations and empirical results, we find that moving to ROI-based planning usually improves agreement over priorities as past studies have claimed. However, the range of improvement we find spans almost complete improvement to very little change. Sometimes we even find that moving to ROI-based planning exacerbates disagreements over priorities. Moving to ROI-based planning in conservation offers many advantages, but our results suggest conservation organizations still need to consider carefully the ecological goals of their protection efforts and how these should be quantified when using ROI-based approaches.

## Results

### Simulations

**Fig. 2:**
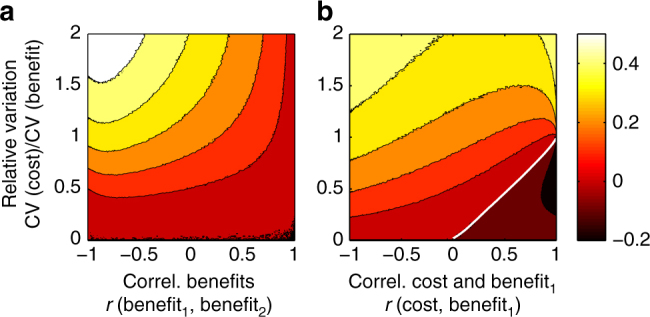
Simulation results showing the mean change in agreement levels when moving to ROI methods. The color map values show the mean change in agreement levels between two budget allocations when ranking candidate sites for protection based on ROI (benefit/cost) vs. when ranking them based on ecological benefits only, i.e., it shows ROI Agreement−Benefit Agreement. The mean change in agreement levels across 2000 replicate simulations is shown for each combination of correlation and relative variation parameters. Lighter colors indicate larger positive changes, for which moving to ROI prioritization results in a larger improvement in agreement levels over what locations should be priorities. The change in agreement level is shown as a function of the relative variation in cost of protection vs. one metric measuring the ecological benefit of protection (vertical axes) and as a function of the correlation (Pearson's) between **a** the two ecological benefit metrics while cost is assumed independent or **b** one ecological benefit metric and the cost of protecting each site while the other benefit metric is assumed independent. Values below the thick white contour in **b** are negative indicating disagreements over priorities are made worse by moving to ROI-based methods. A small number of negative values also occur in the lower right corner of **a** as well but not enough to support a stable contour

We first used simulations to examine our hypotheses (Table 1) about when greater agreement levels over priorities could be expected with ROI-based prioritizations. We generated simulated data on the ecological benefits and economic cost of protecting sites while controlling the relative variation of cost and benefit, covariation between the benefit functions, and covariation between benefit and cost. The dependent variable (indicated by the color map) in Fig. 2 shows the difference in agreement levels between two prioritizations made using an ROI approach and the corresponding prioritizations when based on ecological benefits only; i.e., ROI Agreement−Benefit Agreement in Fig. 1.

**Table 1:** Hypotheses regarding when moving to ROI methods will improve agreement levels

Hypothesis: ROI Agreement>Benefit Agreement when:	Simulation results	Test statistic for case study
H_1_: cost data are more variable than benefit data (larger coefficient of variation).	Supported	*β*_1_ = 0.19, 95% CI = 0.11, 0.28, *p* < 0.001, *n* = 55
H_2_: the two benefit metrics *B*_1_ and *B*_2_ are weakly or negatively correlated.	Supported	*β*_2_ = −0.41, 95% CI = −0.58, −0.24, *p* < 0.001, *n* = 55
H_3_: the benefit of protecting sites is negatively correlated with the costs involved.	Supported for most of parameter space	*β*_3_ = −0.09, 95% CI = −0.37, 0.19, *p* = 0.531, *n* = 55

Hypotheses regarding when agreement levels for ROI-based prioritizations will be larger than agreement levels for prioritizations based only on ecological benefits. We examined these hypotheses with simulations and multiple regressions tests applied to case study data. Test statistics in column 3 are based on estimating multiple regression equation in Eq. (1) and relying on one-tailed, permutation tests to evaluate significance

*ROI* return-on-investment, *CI* confidence interval

Moving vertically across the contours in Fig. 2, we see improvement in agreement levels from accounting for cost is greater when cost data are more variable relative to ecological benefit data (hypothesis H_1_ in Table 1). Moving horizontally from right to left in Fig. 2a, we see that the weaker or more negative the correlation between two ecological benefit metrics, the larger the improvement in agreement levels between two prioritizations when accounting for cost (H_2_ in Table 1). Finally, moving horizontally from right to left in Fig. 2b, we see that the weaker or more negative the correlation between benefit and cost, the greater the improvement in agreement levels between prioritizations that account for cost delivers (H_3_ in Table 1). The exception to this latter signal occurs when cost data are also much more variable than benefit data. In this case, we see some evidence of a unimodal relationship, with greater improvement in agreement levels at intermediate correlation between cost and benefit.

Perhaps more surprising is our finding from the simulations that moving to an ROI framework will heighten disagreements between conservation prioritizations for some distributions of benefit and cost (Fig. 2a, b). Conditions under which accounting for cost appears to make disagreements over priorities worse are the opposite of those highlighted by hypotheses H_1_–H_3_. Specifically, accounting for cost appears most likely to worsen disagreements over priorities when benefit data are more variable than cost data; alternative benefit metrics are already positively correlated; and cost and benefit are also positively correlated. If we ask whether a particular parameter combination sometimes results in increased disagreement when ranking based on ROI, instead of asking whether this will happen on average, then we find a much larger region of parameter space in both panels can give rise to negative results (Supplementary Fig. 1). The possibility that moving to ROI-based planning could increase disagreement levels over priorities has been ignored by all previous studies.

### Case study

For the case study, we focused on protected areas established by TNC, a land trust, to protect forest ecosystems in the central and southern Appalachians. All of our study sites were actually protected. However, we consider a hypothetical scenario examining what would have happened if TNC had not had sufficient resources to protect all of the sites and instead had to prioritize from within them to identify a subset they felt presented the highest priority for protection. By examining TNC’s conservation planning documents, we identified 11 ecological benefit metrics relevant to TNC’s goals for these forest ecosystems (Table 2), allowing comparison of prioritizations based on 55 unique pairs of benefit metrics. These benefit metrics describe various aspects of forest composition, structure, and condition, and the contribution that protecting different sites makes to protecting species and to reducing patterns of forest fragmentation on the landscape. We quantified these benefits through field surveys and GIS analyses. Some benefit metrics quantify ecological attributes of protected parcels themselves and others integrate characteristics of the landscape surrounding protected sites. For cost data, we focused on the upfront acquisition cost to TNC involved in protecting each site.

**Table 2:** Choice of conservation goals and metrics to estimate progress towards these goals

Conservation goal	Ecological benefit metric	Range (IQR)	No. sites, set in Fig. 4
Protect species	No. modeled vertebrate distributions overlapping the site (from 52 total).	23/28/30	*n* = 96, A
	Irreplaceability for vertebrate distributions overlapping the site (i.e., proportion of integer programming solutions that include the site)	0.11/0.13/0.16	*n* = 96, A
	No. of tree species found onsite	16/21/24	*n* = 23, C
	Irreplaceability for the set of tree species found on site	0.02/0.15/0.41	*n* = 23, C
	Modeled increase in expected richness of target species near the site if site is protected	0.001/0.004/0.024	*n* = 46, B
Improve or maintain the structure and condition of forest ecosystems	Mean tree size (DBH)	21.3/24.2/26.7 cm	*n* = 23, C
	Mean percentage canopy cover	82.7/87.8/92.1%	*n* = 23, C
	Mean area of survey plot not covered with invasive plants as a percentage	92.1/98.6/99.6%	*n* = 23, C
Reduce forest fragmentation on the landscape	Change in effective mesh size for protected habitat if parcel is protected	0.1/1.3/9.2	*n* = 96, A
	Percentage of protected land surrounding the parcel	3.7/10.0/16.6%	*n* = 96, A
	Area of protected parcel	8.7/27.9/104.1 ha	*n* = 96, A

Range is presented as IQR (25th/50th/75th percentiles)

*IQR* inter-quartile range, *DBH* diameter at breast height

As expected, we observed shifts in relative priority among sites when including costs into ROI-based approaches. For example, when prioritizing among sites based on ecological condition as estimated using the percent of survey plots not covered by invasive plants, sites that had 99% or more of their area not invaded (10 out of 23 sites) looked like good candidates for protection before considering economic costs. However, these sites were often more expensive. When prioritizing based on ROI, five of those sites that had 99% or more area not invaded were ranked among the 10 worst deals, and the top 10 deals included sites with as little as 83.6% area not invaded. These values apply when focused on our invasive plant metric, and the particular prioritizations obtained and extent of change in those priorities when moving to ROI will depend on the specific benefit considered.

Our hypotheses examine how covariation between benefit metrics, covariation between cost and benefit, and relative variation in cost and benefit (our predictors) affect any change in agreement levels over priorities when conservation organizations rely on different benefit metrics and use ROI-based methods (Table 1). For the case study, we summarize these patterns in predictors in Table 3. Patterns of association between pairs of benefit metrics range from strong negative correlations to strong positive correlations. Across protected areas, cost is much more variable than some benefit metrics and much less variable than others. Correlations between cost and each benefit metric sometimes show a positive correlation and sometimes a negative correlation. These patterns indicate that the case study provides a wide exploration of possible parameter space.

**Table 3:** Summary of benefit and cost distributions and change in agreement levels

	Protect species					Maintain forest condition			Reducing forest fragmentation		
	No. of tree spp.	Irreplace. tree spp.	No. of vert. ranges	Irreplace. vert. spp.	Δ*E* (no. of rare spp.)	Tree size (DBH)	Canopy cover (%)	Not invaded (%)	Δeffect. mesh size	Propn. protect.	Protect. area size
*N*	23	23	96	96	46	23	23	23	96	96	96
No. of tree spp.		0.51***	0.84***	0.81***	−0.89***	0.52***	0.36*	-0.36*	**0.06**	**0.08**	−0.87***
Irreplace. tree spp.	*0.44*		0.42**	0.51***	−**0.39**	0.39**	**0.14**	−**0.25°**	−**0.08**	−**0.01**	−0.42**
No. vert. ranges	*0.00*	*0.44*		0.90***	−0.75***	0.39**	**0.28°**	−**0.24**	−0.14*	−**0.06**	−0.94***
Irreplace. vert. spp.	*0.00*	*0.44*	*−0.05*		−0.76***	0.41**	0.30*	−**0.27°**	−**0.13°**	−**0.04**	−0.91***
Δ*E* (no. of rare spp.)	*0.94*	*0.93*	*0.30*	*0.37*		−**0.11**	**0.50°**	**0.00**	−**0.08**	−**0.02**	0.75***
Tree size (DBH)	*0.41*	*0.52*	*0.41*	*0.41*	*0.93*		0.45**	-0.50***	**0.21**	**0.17**	−0.40**
Canopy cover (%)	*0.26*	*0.68*	*0.26*	*0.26*	*0.94*	*0.29*		**-0.15**	**0.10**	**0.12**	−**0.26°**
Not invaded (%)	*0.88*	*0.70*	*0.88*	*0.88*	*0.94*	*0.96*	*0.99*		−**0.18**	−**0.13**	**0.27°**
Δ effective mesh size	*0.30*	*0.28*	*−0.10*	*0.94*	*0.22*	*0.04*	*0.12*	*0.49*		0.48***	0.14*
Propn. protected	*0.27*	*0.25*	*−0.04*	*0.02*	*0.06*	*0.05*	*0.26*	*0.50*	*−0.02*		**0.06**
Protected area size	*0.97*	*0.78*	*0.31*	*0.30*	*−0.17*	*0.89*	*0.89*	*0.60*	*−0.19*	*0.17*	
$\frac{{{\mathrm CV}_C}}{{{\mathrm CV}_B}}$	0.78	0.49	0.92	0.76	1.04	4.51	7.69	8.50	0.27	1.98	0.94
*τ (B,C)*	−0.57***	−0.41**	−0.63***	−0.62***	0.64***	−0.34*	−0.05	0.32*	0.07	0.09	0.65***

Italic entries show ROI Agreement−Benefit Agreement when relying on a given pair of ecological benefit metrics; bold entries show correlation between pairs of ecological benefits (Kendall’s *τ*); second to last row shows ratio of coefficient of variation of cost over coefficient of variation of benefit; last row shows *τ(B,C)* = correlation between cost and benefit (Kendall’s *τ*). Significance symbols for correlations shown for bold entries: °significant at *p* < 0.10; *significant at *p* < 0.05; **significant at *p* < 0.01; ***significant at *p* < 0.001

*DBH* diameter at breast height, ROI return-on-investment

Table 3 also shows the ROI Agreement−Benefit Agreement we find when following the protocol in Fig. 1. These values provide our response variable. Results can be interpreted similarly to Fig. 2. If there were complete agreement over priorities when moving to ROI despite their having been no agreement over priorities when evaluating sites based on benefit only, we would obtain a value of 1; if there were no improvement in agreement levels, we would obtain a value of 0; and if accounting for cost in an ROI framework worsened disagreements over priorities, we would obtain a negative value. For most pairs of benefit metrics, accounting for cost increased agreement levels over what sites should be priorities. The magnitude of the improvement ranged from being negligible to almost complete. But, as predicted in the simulations, we also observe some negative values where accounting for cost decreased agreement over priorities.

**Fig. 3:**
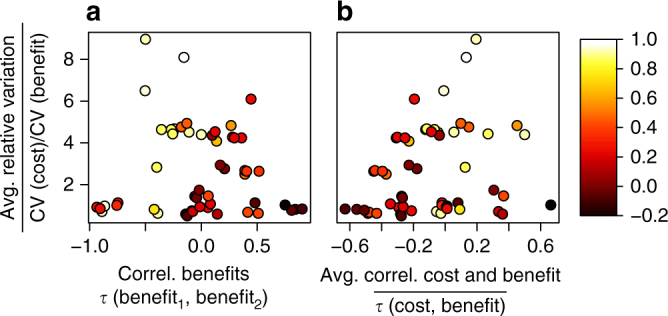
Case study results showing the change in agreement levels when moving to ROI methods. Case study results showing change in agreement levels when moving to ROI-based prioritization (ROI Agreement−Benefit Agreement) for 55 pairs of benefit metrics. Change in agreement levels is shown as a function of (**a**, horizontal axis) the correlation between the two benefit metrics, (**b**, horizontal axis) the correlation between each benefit metric and cost, and (**a** and **b**, vertical axes) the relative variation in benefit and cost, where the latter two measures are averaged across the two benefit functions and Kendall’s *τ* is used to assess correlations. Each point corresponds to a pair of benefit metrics. Lighter shading corresponds to larger improvements in agreement levels

In Fig. 3, we illustrate the same results as Table 3 in a way more comparable to the simulation output. Small changes to a few values in the table arise (e.g., the coefficients of variation (CV) ratio) reflecting different sample sizes available when focusing on particular pairs of benefit metrics. Figure 3 suggests that trends in results for the case study may operate in the same direction as those for the simulations (Fig. 3), something we tested using multiple regression. We found strong support for Hypotheses 1 and 2; relationships were highly significant and in the anticipated direction (Table 1). Accounting for cost led to a greater improvement in agreement levels over priorities when cost data were more variable than ecological benefit data (in Eq. [1] coefficient *β*_1_ = 0.19, 95% confidence interval (CI) = [0.11,0.28], *p* < 0.001, *n* = 55; Fig. 3a, b) and when the ecological benefit metrics were weakly or negatively correlated with each other (coefficient *β*_2_ = −0.41, 95% CI = [−0.58,−0.24], *p* < 0.001, *n* = 55; Fig. 3a). However, we found no support for Hypothesis 3 regarding the role of covariation between the cost and benefit data (Fig. 3b).

## Discussion

Differences over the objectives of conservation or over how progress towards these goals is quantified can result in disagreements over which locations should be priorities for future protection and may impede the development and implementation of conservation plans. However, some authors have argued that these disagreements may be over-stated, because as conservation organizations increasingly turn to ROI-based approaches for spatial prioritization, variation in the economic cost of protecting sites will moderate the influence of contrasting ecological benefit metrics^33,34^. We examined this claim using simulations and case study data from protected areas in the eastern US. We sought to identify when and by how much moving to an ROI framework would enhance agreement between the priorities one would arrive at if valuing different aspects of biodiversity. Our approach and findings may provide a useful template for thinking about how incorporating still other factors, such as threat and opportunity, into ROI-based prioritization will impact debates over the ecological goals driving protection efforts.

In most cases (84% of cases examined), agreement over priorities improved when moving to an ROI framework as Bode et al.^33^ suggested. Yet we observed the full range of variation in the degree of improvement. For some pairs of benefit metrics, there was little agreement when prioritizing based on ecological benefits only, but almost full agreement when taking an ROI approach. For other benefit metrics, we observed very small changes in agreement levels only. Our analyses indicate when we can expect larger improvements. Specifically, our results indicate that how large an improvement in agreement one can expect with an ROI approach is influenced by how variable cost data are relative to the ecological benefit data used and how well correlated the ecological benefit metrics involved are to begin with.

The simulations suggested a more complicated role for the correlation between ecological benefit and cost. For most of parameter space, the simulations suggested that agreement levels between two prioritizations would be improved by a greater amount if cost and benefit were negatively correlated, although there was evidence of some unimodality for very high positive correlations. When we tested for an effect of benefit and cost correlations in the case study, we found it was not important. There are, of course, many reasons why the results of the simulations and case study may differ. For example, the simulations made strong assumptions about the underlying data distributions that may not apply to some variables in the case study. The importance of the correlation between ecological benefit and economic cost is something that has been highlighted before in studies that focus on a single ecological benefit metric only and that examine how moving to an ROI framework changes conservation outcomes for that one metric^44,45^. In Fig. 1, this is akin to scoring agreement levels between the first prioritization in the left-hand, benefit-only column prioritizing sites using *B*_1_ and the first prioritization in the right-hand ROI column prioritizing sites using *B*_1_/*C* without reference to *B*_2_ or alternative choices of benefit metric, and is illustrated by our summary of the change in rankings when using invasive plant cover as an indicator.

Arguably the most surprising result of our work is our finding that in some situations moving to an ROI approach will exacerbate disagreements over which locations should be priorities for protection. This finding contrasts starkly with previous writings on the topic^33,34,36–39^ and warrants scrutiny in future ROI-based studies. Instances where moving to an ROI approach would make disagreements over future priorities worse arise in both the simulations and for a range of benefit metrics in the case study. The benefit metrics that were involved in instances in which agreement levels worsened under ROI-based planning in the case study tended to be ones that were more variable than cost and where the two benefit metrics were positively correlated to begin with. For example, around half of the instances where we obtained a negative value involved our change in effective mesh size measure, the most variable benefit metric that we used.

We necessarily made a number of choices with our analyses. For example, in the case study, we focused on a set of sites that had actually been protected and explored a hypothetical scenario in which budget restrictions meant only a subset of these sites could receive protection. Had we included all land parcels in the region, instead of only those that were particularly interesting to the conservation organization, we would likely have observed different benefit and cost distributions^23^. Also with our analyses, we explored a range of ecological benefit metrics chosen to reflect different conservation goals that motivated protection of the case study sites (e.g., protecting species vs. reducing habitat fragmentation) and different ways of quantifying progress towards these goals. Yet many other choices regarding potential benefit functions and how these could be quantified would have made sense. For example, many organizations prioritize areas based on ecosystem service benefits instead of biodiversity benefits. Similarly, we focused only on acquisition cost and not on other types of cost involved in protecting these sites (e.g., ongoing stewardship costs^46^).

How might other choices about benefits and costs have affected our results? The benefit and cost metrics we use, despite being narrowly focused on conserving forest ecosystems, span a wide range of the possible parameter space involved (Table 3 and Fig. 3). Some are positively correlated with one another and some negatively. Some benefits are much more variable than costs and some much less so. Where we might expect major changes in findings if moving to other kinds of benefit metrics or costs is if those alternative choices ended up sampling a more limited portion of the possible parameter space. For example, some ecosystem service benefits are thought to trade-off against one another^47–49^, meaning when considering such benefits we might be working on the left-hand side of Fig. 3a where the potential improvements in agreement levels from adopting ROI approaches are greater. By thinking through what covariation structures are likely in different contexts in this way, our simulation results can be used to identify what change in agreement levels can be expected.

We focused on ranking approaches as are commonly used to prioritize conservation projects^50^. After using an ecoregional planning process to decide broad regions in which to work, conservation organizations often use ranking methods to evaluate which specific projects within priority regions to pursue. But rather than focus on one benefit at a time as we have done here, an organization would typically consider a range of ecological benefits, perhaps combining them using weights derived from participatory exercises with board members, partners, and stakeholders or through other means^31,51^. How would our results change had we focused on weighted combinations of benefits? Two distinct situations seem relevant. First, were we to assume that all benefit metrics were equally plausible, then the primary effect of combining benefits would be to suppress variation in Fig. 3 through averaging, leading to a smaller volume of parameter space being covered. This would temper the outcomes, producing fewer large improvements when using ROI-based methods and fewer instances where the net improvement was negative. Alternatively, we could have situations where there is agreement over one or two benefits of interest, but disagreement over what additional benefits to include. For example, an organization might value highly irreplaceable communities but also want to assign some value to the ecological condition of forested ecosystems being protected, while not agreeing over just how forest condition should assessed. In this instance, we would expect the starting benefit functions to be more positively correlated than the average we observed (i.e., shifted towards the right-hand side of Fig. 3a; Supplementary Note 1), increasing the likelihood that incorporating costs in ROI-based planning would exacerbate disagreements over which projects to prioritize.

How well conservation organizations agree over priorities determines how effectively they can partner with one another^52,53^. It also determines how well-coordinated conservation actions led by different units within a large conservation organization will be, e.g., state chapters within TNC^54–56^. But there are many ways to define and quantify the ecological goals of conservation, something that has led to disagreements over what locations should be priorities for protection^9,12,57^ and has stimulated research into consensus building methodologies^58–60^. As conservation organizations incorporate additional considerations into their conservation planning, like the cost of protecting different sites, some authors have argued that past disagreements about which aspects of biodiversity to focus on will become less relevant. Our analysis suggests a less rosy and more nuanced picture. While we often find increased agreement over prioritizations when accounting for cost, how large an improvement in agreement varies widely. Moreover, in some instances disagreements will actually be heightened, rather than eased, by moves to account for costs in conservation planning. Incorporating a fuller set of considerations, like economic costs, into conservation planning is worthwhile for many reasons, but doing so will not spare conservation organizations from evaluating carefully what their conservation objectives are and how progress towards meeting them should be quantified.

## Methods

### Protocol

Our analyses follow a standard protocol (Fig. 1). First, we identify the set of sites that are candidates for protection and available conservation budget that limits how many of these sites can be protected.

Next we quantify the agreement in priorities that would result from considering benefits only. To do this, we estimate two different ecological benefit metrics for each site. An ecological benefit metric scores the contribution that protecting a given site would make towards meeting the conservation organization’s overall objective. We then prioritize the subset of sites offering greatest improvement in the conservation organization’s objective according to each benefit metric that is within budget. This should give two subsets of sites, one prioritized relative to each benefit metric. Finally, we quantify the agreement between these two prioritized subsets of sites.

The third step is to quantify the agreement in priorities that would result from considering ROI. We take the first benefit metric and for each candidate site for protection, we divide its benefit score by the cost of protecting the site. This provides an estimate of the ROI protecting the site offers when evaluated against this benefit metric. We do the same for the other benefit metric. We then prioritize the subset of sites recommended for protection using ROI with each benefit metric and quantify agreement between the two resulting ROI-based prioritizations.

The last step is to compare the level of agreement between the two prioritizations obtained from the benefit-only prioritizations with the level of agreement between the two prioritizations obtained from the ROI-based prioritizations.

In implementing this protocol, there are different ways that we could prioritize sites for protection. We take the simplest approach of ranking sites either based on benefits or ROI and prioritizing those ranked highest until the budget is exhausted. Often acquisition of the last site prioritized would exceed the budget and we then assume the next best, affordable site is purchased instead.

There are also different ways to evaluate the agreement between two prioritizations. Following Bode et al.^33^, we report how concordant two budget allocations are. Specifically, we calculate the proportion of the overall budget allocated under each prioritization to the same set of sites (Fig. 1). We refer to this measure as Benefit Agreement when the prioritization considers the ecological benefit metrics alone and ROI Agreement when it is based on the ratio of the benefit metrics to the cost of protecting each site.

### Hypotheses

Our analyses seek to identify when accounting for cost will improve agreement between prioritizations that use different benefit metrics, i.e., when is ROI Agreement>Benefit Agreement? The answers to this question depend on the distributions of the two ecological benefit metrics and the distribution of conservation cost. We test the following hypotheses:

H_1_: “ROI Agreement>Benefit Agreement” when cost data are more variable than benefit data (larger coefficient of variation), because this would give the shared covariate in the ROI prioritizations (cost) more chance to dominate. If cost is more variable than both benefit metrics, then we expect the improvement in agreement levels to be even larger.

H_2_: “ROI Agreement>Benefit Agreement” when the two benefit metrics *B*_1_ and *B*_2_ are weakly or negatively correlated with one another, because under these conditions there is more room for improvement in agreement between prioritizations. In contrast, if the benefit metrics are strongly positively correlated to begin with, then we would already expect very good agreement between prioritizations based on benefits-only, leaving little scope for including cost to enhance agreement.

H_3_: “ROI Agreement>Benefit Agreement” when the benefit of protecting sites is negatively correlated with the cost involved. In this circumstance, the positive relationship between one ROI measure, say *B*_1_/*C*, and 1/*C* is made stronger, because of the assumed negative correlation between *B*_1_ and *C* in the hypothesis. This strengthens the association between *B*_1_/*C* and *B*_2_/*C*, because the latter is also positively associated with 1/*C*, suggesting that agreement between the two ROI prioritizations should be enhanced. The case for improved agreement should be stronger if the second benefit metric *B*_2_ is also negatively correlated with cost.

Other statistical parameters also determine the distributions of benefit and cost. However, we see little a priori reason to support particular hypotheses about their role. For example, it is not clear how the average value of each benefit metric should affect agreement levels, nor the relative variation of the two benefit metrics when compared to one other. While we anticipate that the average cost of protecting sites will be important, the same signal can be recovered by varying the budget (Supplementary Fig. 2).

### Simulations

We first explored these hypotheses using simulations that focused on relative variation of cost and benefit, covariation between the benefit functions and covariation between benefit and cost. We kept all other aspects of the simulations as simple as possible. Specifically, we assumed the three distributions were normal and of equal mean. When focusing on the covariation between one benefit metric and cost or between the two benefit metrics, we assumed the remaining variable was independent of the other two. We split the parameter domain shown in each panel of Fig. 2 into a 240 × 240 parameter grid and ran 2000 replicate runs for each grid point (giving over 230 million simulations in total). Figure 2a, b shows average outcomes across the 2000 replicates for each parameter combination and Supplementary Fig. 1a, b shows the 5th percentile. For each replicate, 100 sites are assumed available for protection and a conservation budget is assigned that is 30 times the average cost of one site.

### Case study

**Fig. 4:**
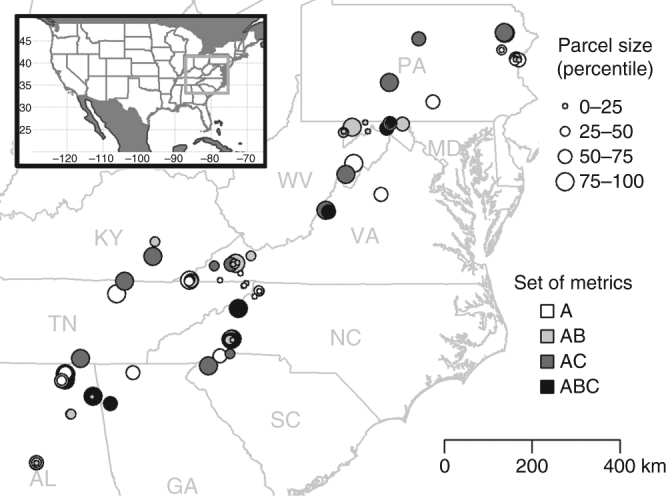
Map of 96 parcels protected by The Nature Conservancy (TNC) used in this study. The size of circles roughly indicates parcel size categories (0–25 percentile: <8.1 ha; 25–50 percentile: 8.1–27.2 ha; 50–75 percentile: 27.2–98.0 ha; 75–100 percentile: >98.0 ha). Size categories are for illustration only—continuous area was used in analyses. Color coding indicates which ecological benefit metrics were available for each site. Five ecological benefit metrics were available for every parcel (set A), species of conservation concern are known to occur near 46 parcels (set B) and field derived benefit metrics were based on a survey of 23 parcels (set C)

We then tested our hypotheses using cost and benefit data for a sample of protected areas acquired by TNC to conserve forest ecosystems in the central and southern Appalachian Mountains (Fig. 4). All of our parcels are located in three TNC ecoregions: the Southern Blue Ridge, Cumberlands and Southern Ridge and Valley, and Central Appalachian Forest and were protected at least partly to protect forested ecosystems. We focus on parcels that TNC acquired between 2000 and 2009. We focus on properties acquired under a fee simple arrangement, meaning the full fee title was acquired, as opposed to properties protected using easements that cover only a subset of the property rights involved^61^. We exclude a small number of sites that were fully donated to TNC for which the acquisition cost was zero. By excluding these sites, we offer a conservative test of our central question, because fully donated sites would automatically be prioritized by an ROI approach regardless of the ecological benefit metric under consideration.

### Ecological benefit metrics

We examined TNC’s regional conservation plans^62^ as well as internal documents outlining the ecological rationale for each acquisition. We also consulted with TNC staff involved in acquiring and managing the sites. From this process, we identified multiple conservation goals that informed the decision to acquire these parcels and various metrics that could be used to quantify progress towards these goals. We arrived at a set of 11 ecological benefit metrics that we used in our analyses (Table 2), including some that address characteristics of the protected sites themselves and others that integrate data on the surrounding landscape. We were thus able to compare prioritizations based on 55 unique pairs of benefit metrics.

We quantified five ecological benefit metrics examining of forest composition, structure, and condition through field surveys on 23 of the protected parcels between May and September 2013 (set C in Fig. 4 and Table 2). Surveyed parcels were distributed across the full spatial domain. These sites also spanned the range of variation found within the full set of parcels, although sites with an area of <20 ha were excluded from field surveys as they were too small for the sampling design. We visited 20 survey points on each of these 23 protected areas. We identified survey points based on randomly allocated GIS locations within the forested area on each parcel. We stratified the random sampling such that 10 points fell within a 100 m buffer parcel boundary and the remainder in the interior. Random points were not permitted within 30 m of each other. At each survey point, we identified the 10 nearest trees with stem radius >10 cm (4600 individual trees), took a diameter at breast height (DBH) measurement of the size of each, and estimated percent canopy cover using a densiometer (reported as percent closed). We also examined the cover of invasive species in the herbaceous understory. We delineated a 5 m radius circular plot (~79 m^2^) around the survey point and focused on the percentage of this area covered by the highest ranked listed invasive plants for each state. In our analysis, we used the percentage of area not covered by invasives as the relevant metric. Using the uninvaded area in this way means that larger values indicate potentially higher ecological benefits as is the case for other metrics we report. For the benefit metrics in Table 2, we used total tree species at the parcel scale and we averaged the DBH measurement, canopy cover, and percentage of the circular plot area that was uninvaded across the survey points for each parcel.

For the tree species data, we also developed an irreplaceability metric to use in addition to considering total richness found on a protected area. Irreplaceability is a site-specific summary statistic obtained from solving an integer programming problem to identify sets of protected sites that, when taken together, allow the most species to be protected^63^. Irreplaceability is defined to be the proportion of solution sets that contain the focal site and protect as many species as possible given a certain budget. We obtained irreplaceability estimates by formulating a maximum coverage problem^64^, solving for near-optimal solutions using a genetic algorithm^65^, and calculating the frequency with which each site occurred in solution sets covering at least 95% of the maximum number of protected species.

We quantified the remaining benefit metrics through GIS analyses for all sites (set A in Fig. 4 and Table 2). TNC’s goals for acquiring these sites include reducing overall habitat fragmentation patterns on the wider landscape. To quantify this, we focus on three different benefit metrics. First, we included how protecting a given parcel changed the effective mesh size of protected habitat in the landscape. Effective mesh size is conceptualized as the probability that two individuals randomly dropped into the landscape will be located in the same patch. This estimate is rescaled into area units such that a measurement of effective mesh size is similar to average patch size^66^. Change in effective mesh size depends on both the amount and spatial arrangement of habitat. However, the amount of habitat is, if anything, more important than its spatial arrangement for determining the ecological effects of fragmentation^67,68^. Therefore, we also included the amount of protected (Gap Analysis Project status 1–3) habitat nearby. For both effective mesh size and the proportion of protected habitat, we defined the surrounding landscape to be a 5 km radius buffer around the centroid of the parcel that TNC acquired—see below for a discussion of this choice of distance. Finally, we included the area of the protected parcel itself, something highlighted in numerous studies as being ecologically important for reducing fragmentation as well as for providing many other ecological benefits and something commonly highlighted in TNC’s own planning documents. We estimated these indices using ArcGIS 10.3^69^ and FRAGSTATS^70^.

As well as protecting forested ecosystems, TNC also aims to protect biodiversity. In developing metrics tied to biodiversity, we included one metric focused on known occurrences of target species, something commonly highlighted in internal TNC documents summarizing why each parcel was a priority for acquisition. The TNC ecoregional plans for the three ecoregions identify a set of species as conservation targets. We developed a metric to identify parcels that would offer the greatest scope for improving the probability of persistence of these target species if protected. We downloaded survey records for the target species from USGS’s Biodiversity Information Serving Our Nation database (accessed August 2013)^71^ for a 5 km equal area buffer around the centroid of each parcel. This distance was chosen to maximize the variation in benefits as well as to match the average distance used to separate survey records into unique element occurrences when movements must occur through unsuitable habitat^72^. This provided records for 65 target species found near our focal sites. To develop our metric, we followed Sutton et al.^23^ and used a simple model to estimate how protecting a given parcel would increase the expected number of known occurrences of a target species that would persist in a buffer around the site. Specifically, we assumed that the change in the probability of persistence for each species occurrence found nearby scaled linearly with the size of the additional area being protected—see Sutton et al.^23^ for more discussion. This is analogous either to assuming management on protected areas will improve the persistence of each species occurrence or that habitat conversion of unprotected sites is such that, over the long term, only sites that are protected will contribute to species persistence. While simple, this approach provides one means of ensuring for persistence vs. coverage trade-offs^13,73^ are included within the set of conservation objectives we consider. Richer predictions would be obtained by including more detail on the life history of the species in question, and spatial heterogeneity either in management efforts within protected areas or habitat conversion threats outside protected areas^74^. When including this ecological benefit metric, we chose to exclude parcels with no known occurrences of target species nearby (leaving 46 protected areas; set B in Table 2 and Fig. 4), to avoid any potentially confounding effects of including one zero-inflated benefit metric alongside the other benefit metrics in our tests of when moving to an ROI framework enhanced agreement levels over priorities.

While known occurrences feature prominently in TNC’s internal documents explaining why specific parcels were protected, modeled species distributions sometimes are preferred in conservation planning^75,76^. Therefore, we also estimated how protected parcels could contribute to protecting species based on their overlap with modeled species distributions. We used data provided by USGS (downloaded June 2015)^77^ on modeled distributions for vertebrate species. Distributions were modeled using species habitat associations, land cover and other relevant factors, e.g., elevation, hydrologic characteristics, tendency of the species to avoid areas of human disturbance, etc.^77^. We used 52 species distributions available for our region^77^. The species in question are predominantly birds (63%) and salamanders (27%). While most of the species are considered secure, 19% are considered imperiled or vulnerable (ranked G2 or G3 by NatureServe^78^). We counted the number of these species whose modeled distributions overlapped each parcel to obtain a local species richness estimate. In addition to this local richness measure for the vertebrate species, we also applied the same process as we used for the data on tree species data to obtain an irreplaceability measure for each site using the vertebrate species distribution data.

Some of these benefit metrics are defined per parcel and others per hectare. We reflected this difference when determining which cost (per parcel or per hectare) to use in ROI calculations. For example, when considering the irreplaceability of a parcel to maximum coverage solutions protecting more species, we use the cost per parcel to estimate ROI. However, when considering the ecological condition of the site using the percent of survey plots not covered by invasive plants, we use the cost per hectare. When conservation prioritizations are conducted based only on benefits, overall area is usually applied as a constraint on the amount of overall conservation activity. In light of this, we focused on benefits per hectare for benefit-only prioritizations. Finally, we include parcel area itself as a possible benefit metric, because of its importance in debates over what makes an ecologically effective protected area.

Importantly, we do not claim any one metric is the correct choice to use for conservation planning in our study region. Instead, our goal in choosing them is only to arrive at a set of contrasting indicators representative of the kinds of ecological benefit measures conservation planners might use to allow us to examine our focal questions.

For our ROI estimation, we need an indicator of the cost of protecting each site. TNC provided data on the upfront acquisition cost they paid to acquire each site. To obtain time consistent estimates of prices, we needed to correct for inflation, something usually done by indexing to inflationary prices rises in related goods. We converted all land prices to 2000 US dollar equivalents using a state level housing price index^79^.

### Prioritization scenario

While all of the parcels included in the case study were actually protected, we focus our analyses on a hypothetical scenario in which we assumed TNC only had sufficient resources to protect a subset of the sites and had to identify those representing the highest priority for protection. By definition, with a scenario like this, the level of agreement between two prioritizations depends on the assumed budget constraint (Supplementary Fig. 2a). If sufficient funding is available to protect all of candidate sites, then there is complete agreement between two prioritizations regardless of the choice of ecological benefit metric used or whether prioritizations were based on benefit-only or ROI-based planning. At the other extreme, if there is only enough funding to protect a single site from within a large set of candidate sites for protection, the agreement between two prioritization methods will often be very low or zero. We chose an intermediate budget level. Specifically, we sought to parse under what set of conditions larger improvements in agreement levels might be expected (Hypotheses 1–3). Thus we chose to run our analyses at a budget level (30% of the budget required to protect all of the sites) where differences between ROI-based planning and benefit-only planning were expressed (Supplementary Fig. 2b). We started each analysis from a pair of ecological benefit metrics (Fig. 1). For each pair, we then identified the full set of candidate sites to be those sites for which both benefit metrics are available (Fig. 4). We defined the focal budget level to be 30% of that needed to acquire this full set of candidate sites. Even at this budget level, where large improvements in agreement levels were observed on average (Supplementary Fig. 2b), we still observed numerous instances in which moving to ROI-based planning decreased the agreement between prioritizations obtained when relying on different ecological benefit metrics.

### Statistical analysis

We use Pearson’s *r* and Kendall’s tau (*τ*) to measure covariation between two benefit metrics or benefit and cost for the simulations and case study respectively. To indicate relative variability between cost and benefit, we rely on the ratio of the relevant coefficients of variation (CV).

We use multiple regression to test Hypothesis 1–3 for the case study. We estimate a model of the form:

1 $${{\mathrm{ROI}}\,{\mathrm{Agreement-Benefit}}\,{\mathrm{Agreement = }}\beta _0 + \beta _1\overline {\frac{{{\mathrm CV}(C)}}{{{\mathrm CV}(B_j)}}} + \beta _2\tau \,({B}_{\mathrm{1}},\,{B}_{\mathrm{2}}) + \beta _3\overline {\tau (C,B_j)} + \varepsilon}$$

where each replicate is given by one of the 55 pairs of benefit metrics, the response variable for each replicate is based on one pass through Fig. 1, predictor variables are derived from the summary statistics for the pair of benefit metrics involved and the site-specific cost, and *ε* is the error term. Each hypothesis is tested by examining the regression coefficient, *β*_*i*_ for *i* = 1,…,3, and we rely on one-tailed, permutation tests to evaluate significance. Hypotheses 1 and 3 depend on relationships between one benefit metric and cost, with the signal being strengthened if both benefit metrics share the same type of relationship with cost. For the regressions, we use the average of the relevant statistic as the explanatory variable across the two benefit metrics. This is delineated in Eq. (1) with the over-bar notation and index *j* = 1,2, which indicates the average is taken across the two benefit metrics. For example, we use $\overline {\tau (C,B_j)}$ = *0.5*(*τ*(*B*_1_ *,C*) + *τ*(*B*_2_ *,C*)) as our explanatory variable when testing Hypothesis 3.

### Data availability

The ecological data that support the findings of this study are available in Dryad (http://www.datadryad.org), with the identifier(s) 10.5061/dryad.415kb.

## Electronic supplementary material

Supplementary Information
