# Supplementary material for: Factoring economic costs into conservation planning may not improve agreement over priorities for protection
Source: Nat Commun. 2017 Dec 21;8:2253. doi: 10.1038/s41467-017-02399-y (PMC5740120; doi:10.1038/s41467-017-02399-y)
Supplement: Supplementary file 1 — Supplementary Information [file 41467_2017_2399_MOESM1_ESM.pdf]

Supplementary Figure 1

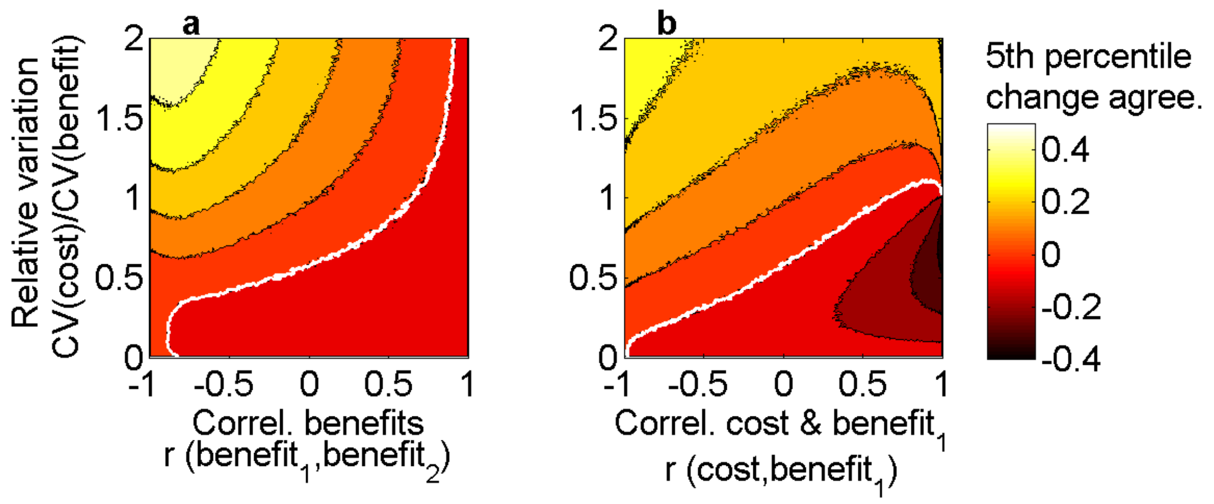

Supplementary Fig. 1. **Simulation results showing the 5th percentile of the change in agreement levels when moving to ROI-methods**.

Equivalent simulation results to Fig. 2 but showing the fifth percentile (instead of the mean) for the change in agreement levels between two budget allocations when ranking candidate sites for protection based on return-on-investment (benefit/cost) versus when ranking them based on ecological benefit only (i.e., ROI Agreement – Benefit Agreement). Lighter colors indicate larger positive changes. Values below the thick white contours are negative. The change in agreement levels is shown as a function of the relative variation in cost versus ecological benefit of protection (vertical axes) and as a function of the correlation (Pearson) between (**a**) the two ecological benefit metrics or (**b**) one ecological benefit metric and the cost of protecting each site.

Supplementary Figure 2

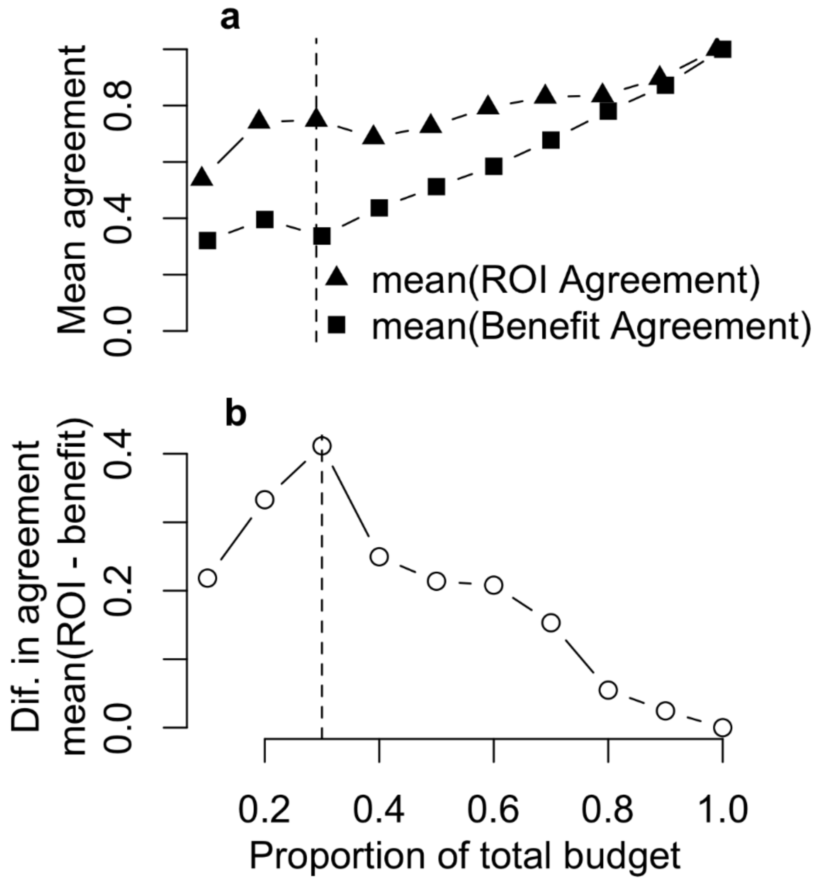

Supplementary Fig. 2. **Change in agreement levels with increasing budget.**

(**a**) shows the increase in mean (ROI Agreement) and in mean (Benefit Agreement) as the proportion of the total budget approaches 1. The means here indicate the average across all 55 pairwise metric comparisons. (**b**) shows the results as mean (ROI Agreement – Benefit Agreement). The dotted line indicates the proportion of the total budget at which our analyses were conducted (0.3). Even at this budget, where, on average, ROI Agreement – Benefit Agreement was large, individual instances occurred where including cost and using ROI-based prioritization actually decreased agreement levels relative to benefit-only prioritization.

Supplementary Note 1

In the Discussion, we describe the effect of repeating our design (Fig. 1) using weighted combinations of benefit metrics. The specific example we mention concerns a conservation organization agreeing that irreplaceability - here we will assume for tree community - is important and also wanting to include some indicator of forest condition but with no agreement over how condition should be assessed. Perhaps one regional office focuses on invasives cover as an indicator of ecological condition while another focuses on large scale fragmentation patterns measured by the change in effective mesh size. This would give rise to two new benefit functions:

$$\widehat{B_1} = w\ Irreplaceability + (1 - w)\ Noninvasive$$

$$\widehat{B_2} = w\ Irreplaceability + (1 - w)\ \Delta\ effective\ mesh\ size$$

The shared variable in these two combination benefit functions (*Irreplaceability*) induces a positive correlation between them. We tested this idea using the example outlined above after dividing each of the starting metrics by their mean value to put them on a comparable axis scale. Including even an equal weight (w = 0.5) induced much stronger, positive correlations between the combination benefit metrics ($\widehat{B_1}$ and $\widehat{B_2}$) than we observed when focusing on the component benefit metrics that they contain (Table 3). Having a more positive correlation would move us to the right in Figs. 2a, 3a and Supplementary Fig. 1a, where using ROI methods is more likely to worsen disagreements over priority projects.
